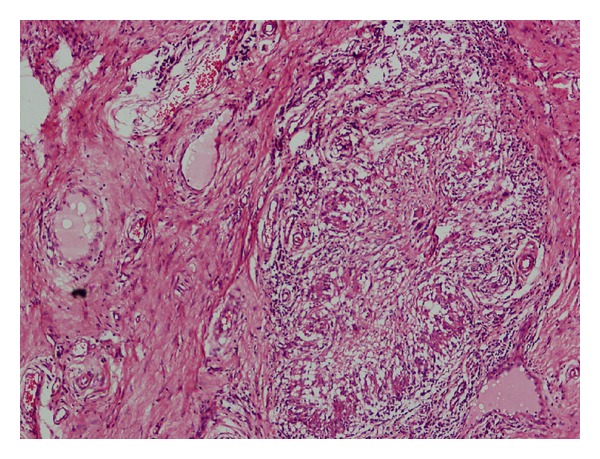# Vulval Elephantiasis: A Case Report

**DOI:** 10.1155/2012/430745

**Published:** 2012-11-01

**Authors:** Harsh Mohan, Bhumika Bisht, Poonam Goel, Geeta Garg

**Affiliations:** ^1^Department of Pathology, Government Medical College, Sector 32-A, Chandigarh 160031, India; ^2^Department of Obstetrics and Gynecology, Government Medical College, Sector 32-A, Chandigarh 160030, India; ^3^Department of Dermatology & Venereology, Government Medical College, Sector 32-A, Chandigarh 160030, India

## Abstract

*Introduction*. Elephantiasis is a chronic manifestation of filariasis; it commonly affects limbs, scrotum, and trunk. Females have lower incidence of filarial infection. Vulval elephantiasis due to filariasis is still rarer. It is difficult to make the diagnosis on histopathology alone, more so in view of the fact that the parasite is usually not identified in tissue sections. Identification of microfilariae in night samples of peripheral blood or seropositivity for filarial antigen is requisite for the correct diagnosis. *Case Presentation*. A young female presented with progressively increasing vulval swelling over a period of two years. The swelling was soft and measured 5 × 6 cm. Other possible differential diagnoses were excluded, and ancillary tests were performed to reach a conclusive diagnosis of vulval elephantiasis on histopathology. *Conclusion*. Vulval elephantiasis due to filariasis is rare. Its diagnosis on histopathology is more often by exclusion. High index of suspicion on microscopic findings and corelation with relevant diagnostic tests are required to reach the correct diagnosis.

## 1. Introduction 

Elephantiasis is a rare disorder involving lymphatic channels of the affected part of the body. It is characterised by gross enlargement of the particular body part. Most commonly, it is caused due to filarial parasites. The prototype of this disease was depicted by involvement of lower limbs. Other areas like trunk, breasts, upper limbs, and external genitalia can also be involved. Genital elephantiasis, also known as esthiomene, is a rare, dramatic end result of lymphatic obstruction. Although mainly associated with filariasis and sexually transmitted diseases such as lymphogranuloma venereum and donovanosis, it could also be an uncommon complication of tubercular lymphadenitis [1, 2]. Filarial elephantiasis of the female genitalia is extremely uncommon and is reported infrequently as isolated case reports [3].

## 2. Case Presentation

An 18-year-old, unmarried female presented with the complaint of low grade fever on and off and a perineal swelling. The swelling had been present for 2 years, slowly increasing to its present size. The patient was a migrant from Azamgarh, Uttar Pradesh, India.

On general physical examination, the patient was afebrile and there was no lymphadenopathy. Local examination revealed a polypoidal growth measuring 5 × 6 cm, arising from the left labium minus. The growth was soft, and the overlying skin showed rugosities. A clinical diagnosis of fibrolipoma was suggested. 

Fine needle aspiration of the growth was performed. Microscopy showed hypocellular smears with an occasional lymphocyte in a proteinaceous background. 

**Figure 1:**
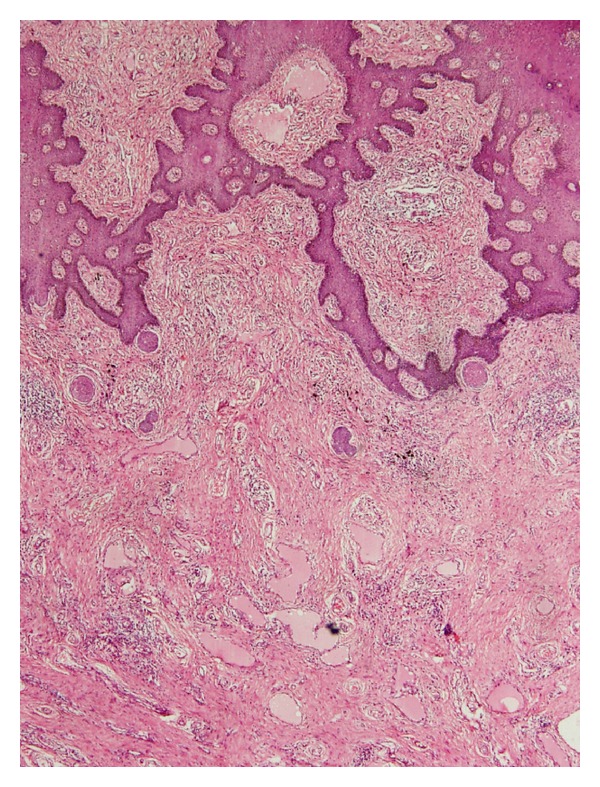


Subsequently, the growth was excised and submitted for histopathological examination. Grossly, it was a single, polypoidal soft tissue mass measuring 5.2 × 3.4 × 2.2 cm. The cut section was grey-white to grey-yellow and firm. Microscopic examination showed a polypoidal lesion covered by acanthotic epidermis. The dermis and subcutis showed dense collagenization and contained numerous dilated lymphatics (Figure 1). In addition, deep dermis showed a few noncaseating granulomas along with foreign body giant cells (Figure 2). There was patchy cellular infiltrate consisting of lymphocytes, plasma cells, and eosinophils.

Ziehl-Neelsen stain for acid-fast bacilli and Grocott's methenamine silver and periodic acid Schiff (PAS) stains for fungus were negative. Buffy coat preparation of the patient's peripheral blood was also examined, but no microfilariae could be seen. The histopathological diagnosis was suggested as vulval elephantiasis, and further workup was suggested.

Subsequent to histopathologic report, filarial antigen serology was done, which was positive. The patient was put on ivermectin, and she responded well.

## 3. Discussion 

Lymphatic filariasis affects 120 million people in more than 80 countries throughout the tropics and subtropics. One-third of those infected live in India, one third in Africa, and the remainder in South-Asia, the Pacific and the Americas [4]. Elephantiasis is an uncommon chronic manifestation of filarial infection, secondary to lymphatic involvement. The death of adult worms provokes acute inflammation and lymphatic dysfunction, and the late effects of the disease, such as elephantiasis, result from superimposed bacterial infection in areas of lymphatic dysfunction [5]. The term elephantiasis was first used by Celsus (30 BC–50 AD). It was also known as satyriasis, sarcocele, or leontiasis. 

The most widespread filarial infection is due to *Wuchereria bancrofti *(98%) and the remaining to *Brugia malayi* (2%) [6]. In India, the state of Bihar has highest endemicity (over 17%) followed by Kerala (15.7%) and Uttar Pradesh (14.6%). Mosquito vector for disease transmission in humans is *Culex quinquefasciatus *for bancroftian filariasis, whereas the genus *Mansonoides* for brugian filariasis [7]. 

Filariasis can present as asymptomatic/subclinical microfilaremia, acute disease characterised by lymphadenitis or lymphangitis, and chronic manifestation characterised lymphedema or elephantiasis. The gender of the individual has an important influence on the incidence of chronic pathology in lymphatic filariasis; males are more commonly susceptible to chronic sequelae than women. The manifestations in descending order of occurrence are hydrocele, followed by elephantiasis of the entire lower limb, the scrotum, the entire arm, the vulva, and the breast. The rare condition of vulval elephantiasis has been reported to be caused by a variety of etiologies like lymphatic filariasis, lymphogranuloma venereum, donovanosis, and tuberculous lymphadenitis. Irrespective of the etiology, the basic process remains the same, that is, permanent obstruction of lymphatic channels → lymphatic stasis → stimulation of growth of fibroblasts → destruction of lymph nodes → lymphedema and elephantiasis [8].

This case presented as a polypoidal mass involving the left labium minus. Typically, elephantiasis diffusely involves the body part affected; exceptionally, lymphedema involves circumscribed region of skin producing a solitary, large polyp, indurated (solid) plaque, papillomatous plaque, pendulous swelling, or large, sarcoma-like mass [9]. 

The histopathological features in this case were suggestive of vulval elephantiasis. Ancillary tests are required to reach a definite diagnosis as in the present case, neither the adult parasite was seen in the tissue examined, nor were the microfilariae demonstrated in the peripheral blood. It is postulated that as long as the adult worms are alive, the lymphatic vessels, though damaged, still remain patent [10]. Death of the worm, however, leads to progressive fibrosis, obliteration of vessels by granuloma, thrombi formation, and extensive perilymphangitis resulting in irreversible lymphatic damage. Therefore, the identification of intact worm in tissue sections is doubtful. 

Fibroepithelial polyp was considered as another possible differential diagnosis. However, absence of loose myxoid stroma, acanthosis of overlying epidermis, and presence of numerous dilated lymphatics ruled out this possibility. Due to finding of granulomas, giant cells, and cellular infiltrate comprising lymphocytes, plasma cells, and eosinophils, the other possibilities that enter into the differential diagnosis include tuberculosis and fungal infection. Presence of noncaseating granulomas, absence of acid-fast bacilli, and heterogenous admixture of cellular infiltrate ruled out tuberculosis. Absence of fungal profiles by special stains excluded possibility of fungal infection.

In conclusion, thorough sampling of the excised tissue to search for the parasite and high index of clinical and histopathological suspicion led to the suggestion of vulval elephantiasis on histopathology. The diagnosis was further supported by positive filarial antigen serology. 

## Figures and Tables

**Figure 2:**